# General anxiety, depression, and physical health in relation to symptoms of heart-focused anxiety- a cross sectional study among patients living with the risk of serious arrhythmias and sudden cardiac death

**DOI:** 10.1186/1477-7525-9-100

**Published:** 2011-11-14

**Authors:** Anniken Hamang, Geir E Eide, Berit Rokne, Karin Nordin, Nina Øyen

**Affiliations:** 1 Genetic Epidemiology Research Group, Department of Public Health and Primary Health Care, University of Bergen, Norway; 2 Center for Medical Genetics and Molecular Medicine, Haukeland University Hospital, Bergen, Norway; 3 Center for Clinical Research, Haukeland University Hospital, Bergen, Norway; 4 Research Group on Lifestyle Epidemiology, Department of Public Health and Primary Health Care, University of Bergen, Norway; 5 Department of Public Health and Primary Health Care, University of Bergen, Norway; 6 Department of Public Health and Caring Sciences, Uppsala University, Sweden

**Keywords:** Anxiety, Depression, Physical Health, Heart-focused anxiety, Long QT syndrome, Hypertrophic Cardiomyopathy

## Abstract

**Objective:**

To investigate the role of three distinct symptoms of heart-focused anxiety (cardio-protective *avoidance*, heart-focused *attention*, and *fear *about heart sensations) in relation to general anxiety, depression and physical health in patients referred to specialized cardio-genetics outpatient clinics in Norway for genetic investigation and counseling.

**Methods:**

Participants were 126 patients (mean age 45 years, 53.5% women). All patients were at higher risk than the average person for serious arrhythmias and sudden cardiac death (SCD) because of a personal or a family history of an inherited cardiac disorder (familial long QT syndrome or hypertrophic cardiomyopathy). Patients filled in, Hospital Anxiety and Depression Scale, Short-Form 36 Health Survey, and Cardiac Anxiety Questionnaire, two weeks before the scheduled counseling session.

**Results:**

The patients experienced higher levels of general anxiety than expected in the general population (mean difference 1.1 (p < 0.01)). Hierarchical regression analyses showed that avoidance and fear was independently related to general anxiety, depression, and physical health beyond relevant demographic covariates (age, gender, having children) and clinical variables (clinical diagnosis, and a recent SCD in the family). In addition to heart-focused anxiety, having a clinical diagnosis was of importance for physical health, whereas a recent SCD in the family was independently related to general anxiety and depression, regardless of disease status.

**Conclusion:**

Avoidance and fear may be potentially modifiable symptoms. Because these distinct symptoms may have important roles in determining general anxiety, depression and physical health in at-risk individuals of inherited cardiac disorders, the present findings may have implications for the further development of genetic counseling for this patient group.

## Introduction

Long QT syndrome (LQTS) and hypertrophic cardiomyopathy (HCM) are cardiac disorders that can cause syncope, palpitations, serious arrhythmias and sudden cardiac death (SCD) [1-3]. This health threat may cause fearful reactions to cardiac-related stimuli and sensations in patients with familial LQTS and familial HCM.

It is likely that this health threat influence not only individuals that are diagnosed with LQTS or HCM, but also their relatives at risk. Familial LQTS and familial HCM are genetic disorders caused by gene mutations inherited in an autosomal dominant fashion. Children, siblings, and parents of affected patients have 50% risk of having the same gene mutation predisposing for LQTS or HCM. The possibility for molecular genetics investigation in affected individuals (patients with a diagnosis) and their relatives (patients at genetic risk) represents a challenge in the genetic counseling session with respect to information, education, and especially psychosocial support, due to the lack of systematic knowledge of how these patients are affected by living with familial LQTS or familial HCM.

While HCM is a quite common genetic disease affecting one in 500 people [4], LQTS affects approximately one in 2500 [1]. LQTS is an ion channel disease leading to a prolonged QT interval with an increased propensity to ventricular tachycardia manifesting as torsade de pointes [5,6]. HCM is defined by the presence of increased ventricular wall thickness or mass, having ruled out hypertension or a valve disease [2]. In addition to the risk of arrhythmia and syncope, HCM can give dyspnoea, chest pain, and exertional angina [7]

The cardiac symptoms manifesting in these patients can lead to proper management of the disease and preventive measures, such as medication (beta blockers for LQTS), devices (implantable cardioverter defibrillators for LQTS and HCM), and lifestyle modifications (restrictions of intense sports for LQTS and HCM) [8]. Because of the reduced penetrance and variable expression of these diseases, a substantial proportion of the individuals will never actually experience manifested disease [9,10]. The management of these disorders is therefore complicated for the caregivers, creating a lot of uncertainty and distress when interpreting signs and symptoms for the individuals at risk [11,12]. In addition, information of being at risk of a possible life-threatening cardiac disorder and experiencing sudden cardiac death in the family may create a burdensome life uncertainty [13].

Research based on patient-reported outcomes in at-risk individuals with familial LQTS or familial HCM is scarce and more is needed in order to understand the impact of living with the risk of serious arrhythmias and sudden cardiac death, also to identify possibilities for intervention. In previous reports, the elevated anxiety and distress levels among individuals with familial LQTS have been measured in parents in relation to genetic test results of their children [14,15]. In adult HCM patients, living with HCM has been reported to be associated with raised levels of anxiety and depression and decreased levels of physical and mental health as compared to the general population [16], while mutation carriers at risk have been found to be no different than the general population. However, experiencing symptoms and having a higher perceived risk of symptoms have been reported to contribute to poorer physical and mental health in HCM mutation carriers [11].

Given the potential serious consequences of both cardiac disorders, heart-focused anxiety may occur in the patients attending genetic counseling. Heart-focused anxiety, defined as a fear of cardiac-related events and sensations based on presumed harmful consequences (i.e. serious arrhythmia, sudden cardiac death) can be measured by the Cardiac Anxiety Questionnaire (CAQ) [17]. Symptoms indicative of heart-focused anxiety is cardio- protective *avoidance *behavior to minimize cardiac symptoms or complications, increased levels of heart-focused *attention *and monitoring of cardiac related stimuli, and *fear *and worries about heart-sensations and functioning. Higher degrees of these symptoms indicate higher degrees of heart-focused anxiety [17-19]. Such fearful symptoms may contribute in raising levels of general anxiety and depression, and influence patient-reported physical health beyond the effects of relevant socio-demographic and clinical variables previously shown to be common confounders of these patient-reported outcomes [11,12,14-16]. In earlier studies, high levels of heart-focused anxiety have been reported in patients with a heart-disease, but also in patients without a heart-disease [18-21], chest pain intensity has been predicted by heart-focused attention and fear in patients with coronary disease [22], and in patients undergoing cardiac surgery, heart-focused anxiety has been shown to be significantly correlated with increased symptoms of anxiety and depression and lower health-related quality of life [20]. In the present population heart-focused anxiety have been found to be higher in patients with a clinical diagnosis of LQTS or a clinical diagnosis of HCM as compared to patients at genetic risk [23]. However, to our knowledge, the role of the distinct symptoms of heart-focused anxiety (avoidance, attention and fear) in relation to general anxiety, depression and physical health has never been investigated in individuals with familial LQTS or familial HCM, thus making this our overall aim. On the issue of how to increase our competence on the LQTS or HCM patients who seek genetic counseling and to address our overall aim, we therefore investigated (i) these patients' level of general anxiety, depression and physical health and compared the scores to expected scores of the general population, (ii) the scores of general anxiety, depression, physical health, and heart-focused anxiety (avoidance, attention, fear) in patients referred because of familial LQTS as compared to the scores of patients referred because of familial HCM, and (iii) the role of avoidance, attention, and fear symptoms in relation to general anxiety, depression, and physical health in the total sample.

It was hypothesized that the patients general anxiety and depression scores would be elevated and that physical health would be poorer compared to the expected scores of the general population, and further that the levels of general anxiety and depression and heart-focused anxiety (avoidance, attention, fear) would be lower, and that the physical health would be better in patients referred for familial LQTS as compared to familial HCM, since HCM patients often exhibit more debilitating symptoms. Finally, it was hypothesized that higher scores of avoidance, attention and fear symptoms would significantly and uniquely be related to (1) higher level of general anxiety, (2) higher level of depression, and (3) poorer physical health. In all models it was expected that the three distinct symptoms of heart-focused anxiety would be significant beyond demographic covariates (gender, age, having children) and clinical variables (clinical diagnosis of either LQTS or HCM, and a recent SCD in the family).

## Methods

### Participants

The participants comprised patients with the risk of serious arrhythmia and sudden cardiac death, because of familial LQTS or familial HCM. Patients with a personal history (with diagnosis) or a family history of LQTS or HCM (at genetic risk), and who were consecutively referred or self-referred to genetic counseling at the medical genetic departments in Bergen, Trondheim, or Oslo during the period 2005 through 2007 were eligible for the study. One hundred and seventy-three patients that were not previously genetic tested were asked to participate in the study. Of these, 35 did not consent to participate and 7 did not return the questionnaire. One did not attend genetic counseling, one did not fill out relevant questions in questionnaire, and 3 patients were not included due to administrative failure, leaving 126 (72.8%) patients included in the analyses.

### Procedure

Participants filled in the questionnaires with information on socio-demographic variables, and measuring general anxiety and depression, physical health, and symptoms of heart-focused anxiety (avoidance, attention, and fear), whereas information about diagnosis was obtained from the medical records. Information about the study and a consent form was mailed to the patient together with the questionnaire 2-4 weeks before the genetic counseling. The participants received one reminder. The study was approved by the Regional Committee for Medical Research Ethics in Western Norway in September 2004.

### Measures

#### General anxiety and depression

The Hospital Anxiety and Depression Scale (HADS) measures anxiety and depression on two subscales; HADS-anxiety (7 items), and HADS-depression (7 items) [24]. A higher score means a higher level of general anxiety or depression (scores ranging from 0-21). It is well suited as a screening tool for general anxiety and depression, also in HCM patients, with a cut-off score of 8 to detect clinical cases [25].

#### Physical health

*The Short Form-36 Health Survey (SF-36) *is a self-report questionnaire that measures health status domains (0 = worst health state; 100 = best health state) on eight sub-scales, where physical functioning, role limitation-physical, bodily pain and general health are mainly considered physical health domains and vitality, social functioning, role limitation-emotional and mental health are considered mental health domains. The physical health domains form the basis to calculate a physical component summary (PCS) that becomes an overall assessment of physical health which includes both functioning and evaluation of one's ability to perform physical activity. The PCS is standardized for the general population with a mean score of 50 and a 10 points standard deviation. The questionnaire is generic and multidimensional, and suitable for administration to large populations and also to patient subgroups. Its purpose is to be a measure of health status or health outcome in cross-sectional and longitudinal studies [26,27]. The SF-36 is a reliable and valid measure across studies all over the world, and the Norwegian version exhibits satisfactory psychometric properties [28].

#### Heart-focused anxiety

*The Cardiac Anxiety Questionnaire *(CAQ) measures heart-focused anxiety in patients with and without heart diseases or cardiac symptoms [17]. It consists of 18 items, and the three subscales; avoidance, attention, and fear may be regarded as the patients' fearful symptoms of heart-focused anxiety to cardiac-related stimuli or sensations based on the belief that they will lead to negative consequences. Each item is rated on a 5-point Likert scale; with higher scores indicating higher levels of heart-focused anxiety. The questionnaire was translated to Norwegian by a professional translator, using a forward and backward translation procedure.

#### Socio-demographic variables and diagnosis of LQTS and HCM

Data were obtained on gender, age, having children, SCD in first or second degree relatives, recent SCD in the family, and clinical diagnosis vs. genetic risk of LQTS or HCM. Recent SCD was defined as cardiac death in a relative in the last year.

#### The general population

Expected scores of general anxiety and depression were calculated based on the normative data from 54,867 subjects aged ≥ 20 years with complete data on the HADS, smoking, and education variables, and without self-reported previous cardiovascular disease [29]who participated in the Nord-Trøndelag Health Study 1995-97 in Norway (the HUNT 2 Study)[30], whereas US physical health norms according to SF-36 norm-based scoring were used, when comparing the physical health scores in the study with a general population [27]

### Statistical analysis

The sample characteristics were summarized by calculating means, standard deviations (SD) for the continuous variables, and by absolute numbers and percentages for the categorical variables. Bivariate analyses were performed with paired samples t-tests when comparing levels of general anxiety and depression in the sample with the expected scores from the general population, with one samples t-test to compare physical health in the sample to US norm scores, and with independent samples t-test when comparing patient groups.

A series of three hierarchical multiple regression analyses were conducted to examine avoidance, attention, and fear entered concurrently, in relation to general anxiety, depression and physical health. Preliminary analyses with Spearman rank correlation was estimated to study the association between variables and to check that the correlation between the independent variables (avoidance, attention, and fear) was not too high to include them as independent determinants in the regression models.

To investigate the ability of the models (which includes avoidance, attention, and fear) to predict levels of general anxiety, depression, and physical health, beyond relevant demographic covariates (age, gender, having children) and clinical variables (clinical diagnosis of either LQTS or HCM, and a recent SCD in the family), the use of a hierarchical multiple regression method was justified. The results were reported as unstandardized regression coefficient (B), standard error (SE) to investigate the relationship of the independent variables to the dependent variables, standardized regression coefficients (beta) to compare the contribution of each independent value, and F-statistics with p-values and determination coefficient with R^2^change were reported to indicate how much of the overall variance is explained by our variables of interest after the effects of relevant socio-demographic and clinical variables. The unstandardized regression coefficient indicates the strength of relationship between a given predictor, and an outcome in the units of measurement of the predictor. It is the change in the outcome associated with a unit change in the predictor, whereas the standardized regression coefficient indicates the strength of relationship between a given predictor and an outcome in a standardized form. It is the change in the outcome (in standard deviations) associated with a one standard deviation change in the predictor, thus it is suitable for comparing the effects of predictors possibly measured on different scales or in different units of measurement.

All tests were two-tailed at the 5% significance level. Data were analyzed using SPSS version 15.0.

## Results

### Sample characteristics

Among the 126 study participants, the mean age was 45 years (SD = 16) and 53% (n = 67) were women. It was found that 70% of the patients (n = 88) had a LQTS family history or were affected clinically with LQTS (familial LQTS group), and 30% of the patients (n = 38) reported a HCM family history or were affected clinically with HCM (familial HCM group). Seventy-eight percent of the patients (n = 98) had children, and 28% of the patients (n = 35) had experienced a SCD in a first or second degree relative, 20% of the patients (n = 25) as recent as in the last year. Of the total sample of patients, 25% (n = 32) had a clinical diagnosis of either LQTS or HCM as opposed to 75% (n = 94) at genetic risk because of family history of LQTS or HCM. The socio-demographic variables of the study population are more extensively described in a recent publication [31].

### Patients level of general anxiety, depression, and physical health as compared to expected scores of the general population

In the present sample, the proportion of patients with clinical HADS scores 8 or greater for general anxiety or depression were 24.6% (n = 31) and 13.5% (n = 17), respectively. Whether the patients were at genetic risk or were diagnosed with LQTS or HCM did not cause significant differences in levels of general anxiety (mean difference -0.1, t (-0.1), p = 0.90 (two-tailed)) and depression (mean difference -0.4, t (-0.5), p = 0.64 (two-tailed)).

Overall, the study group (n = 125) had significantly higher levels of general anxiety as compared to expected scores of the general population (mean difference 1.1, t (3.2), p < 0.01 (two-tailed)); adjusted for gender, age, education level, and smoking status), whereas depression levels were similar to expected scores (mean difference -0.2, t (0.7), p = 0.50 (two-tailed)). Moreover, physical health did not differ significantly from expected scores. However, patients at genetic risk (n = 89) scored better on physical health as compared to expected scores (mean difference 2.3, t(3.0), p < 0.01 (two-tailed)), whereas the patients with clinical diagnosis of either LQTS or HCM (n = 31) showed poorer physical health as compared to expected scores (mean difference -4.5 t(-2.4), p = 0.02 (two-tailed)) (Table 1).

**Table 1:** General anxiety, depression (HADS), and physical health (PCS) in individuals with familial Long QT syndrome (LQTS) and Hypertrophic cardiomyopathy (HCM) as compared to expected scores of general population.

Patient-reported outcomes		n	Study sample	Norwegian expected scores*	p-value
HADS-	Total sample	125	4.9 (4.0)	3.8 (0.5)	**<0.01**
Anxiety	At genetic risk	93	4.9 (3.8)	3.9 (0.5)	**0.01**
(0-21)	With clinical diagnosis	32	5.0 (4.5)	3.7 (0.5)	0.10
HADS-	Total sample	125	3.1 (3.7)	2.9 (0.7)	0.50
Depression	At genetic risk	93	3.0 (3.8)	2.9 (0.7)	0.65
(0-21)	With clinical diagnosis	32	3.4 (3.7)	3.0 (0.8)	0.58
SF36-	Total sample	120	50.6 (8.6)	50.0 (10)	0.48
Physical health	At genetic risk	89	52.3 (7.2)	50.0 (10)	**<0.01**
Summary (0-100)	With clinical diagnosis	31	45.5 (10.4)	50.0 (10)	**0.02**

HADS: Hospital Anxiety and Depression Scale; PCS: SF-36 Physical Component Summary; results are presented as mean (standard deviation), number of participants and p-values.

*Expected scores, based on Norwegian general population, adjusted to the age, gender, education level, and smoking habits distribution in the sample (n = 125)

### Comparisons between patients with familial LQTS and patients with familial HCM

When comparing the patients with familial LQTS to patients with familial HCM, there were no significant differences with regard to level of general anxiety and depression, whereas poorer physical health (mean difference 4.5, t(2.5), p < 0.01(two-tailed)) and higher scores of avoidance (mean difference -0.7, t(-4.1), p < 0.01(two-tailed)), attention (mean difference -0.5, t(-3.6), p <0.01 (two-tailed)), fear (mean difference -0.5, t(-3.3), p < 0.01 (two-tailed)) were found in the latter group.

In the subgroups, patients at genetic risk had higher fear scores in HCM families as compared to in LQTS families, whereas there were no significant differences in the other patient-reported outcomes. Patients with a clinical diagnosis had poorer physical health and higher avoidance scores in HCM families as compared to in LQTS families, whereas significant differences were not found in level of general anxiety and depression, or in attention or fear scores. (Table 2).

**Table 2:** General anxiety, depression (HADS), physical health (PCS), and heart-focused anxiety (CAQ-avoidance, -attention and -fear) scores of individuals with familial Long QT syndrome (LQTS) as compared to individuals with familial Hypertrophic cardiomyopathy (HCM).

Patient-reported outcomes		Familial LQTS	n	Familial HCM	n	p-value
HADS-	Total sample	5.0 (4.1)	87	4.7 (3.8)	38	0.74
Anxiety (0-21)	At genetic risk	5.1 (3.9)	75	4.0 (3.5)	18	0.29
	With clinical diagnosis	4.3 (5.2)	12	5.3 (4.1)	20	0.54
HADS-	Total sample	3.0 (4.0)	87	3.3 (2.9)	38	0.65
Depression (0-21)	At genetic risk	3.1 (3.9)	75	2.8 (3.1)	18	0.76
	With clinical diagnosis	2.6 (4.8)	12	3.9 (2.9)	20	0.35
SF36-	Total sample	52.0 (7.1)	84	47.1 (10.5)	36	**0.01**
Physical health	At genetic risk	52.2 (7.1)	72	52.8 (7.6)	17	0.75
Summary (0-100)	With clinical diagnosis	51.0 (8.6)	12	42.1 (10.2)	19	**0.02**
CAQ-	Total sample	0.7 (0.7)	87	1.4 (0.9)	38	**<0.01**
Avoidance (0-4)	At genetic risk	0.8 (0.7)	75	1.1 (0.7)	18	0.06
	With clinical diagnosis	0.6 (0.4)	12	1.7 (1.0)	20	**<0.01**
CAQ-	Total sample	0.6 (0.6)	88	1.1 (0.8)	38	**0.01**
Attention (0-4)	At genetic risk	0.6 (0.5)	76	0.8 (0.7)	18	0.11
	With clinical diagnosis	0.8 (0.9)	12	1.3 (0.8)	20	0.13
CAQ-	Total sample	1.0 (0.8)	87	1.5 (0.7)	38	**<0.01**
Fear (0-4)	At genetic risk	1.0 (0.7)	75	1.4 (0.7)	18	**0.05**
	With clinical diagnosis	1.3 (1.0)	12	1.7 (0.8)	20	0.34
						

HADS: Hospital Anxiety and Depression Scale; PCS: SF-36 Physical Component Summary; HADS, and Cardiac Anxiety Questionnaire; CAQ are presented as mean (standard deviation) and number of participants

### Correlational analyses

As shown in table 3, there were significant correlation coefficients between pair-wise comparisons of the independent variables (avoidance, attention, and fear) and the dependent variables (general anxiety, depression, and physical health). The most negative and significant correlation coefficient was between avoidance and physical health (r = -0.44). Attention and general anxiety (r = 0.45), and fear and depression (r = 0.45) had the most positive correlation coefficients. Among the independent variables the strongest correlation coefficient was between attention and fear (r = 0.66).

**Table 3:** Spearman correlations between the study variables.

	**1**.	**2**.	**3**.	**4**.	**5**.	**6**.
1. CAQ Avoidance	-	0.43**	0.46**	0.39**	0.44**	-0.44**
2. CAQ Attention		-	0.66**	0.45**	0.37**	-17.5
3. CAQ Fear			-	0.44**	0.45**	-0.37**
4. HADS Anxiety				-	0.68**	-0.27**
5. HADS Depression					-	-0.40**
6. PCS Physical health						-
						
*N*	125	126	125	125	125	120

CAQ: Cardiac Anxiety Questionaire; HADS: Hospital Anxiety and Depression Scale; PCS: SF-36 Physical Component Summary;*p < 0.05; **p < 0.01

### Symptoms of heart-focused anxiety independently related to general anxiety, depression and physical health

Table 4 summarizes the hierarchical regression analysis for general anxiety, depression, and physical health. In terms of general anxiety, the variables (gender, age, having children, clinical diagnosis of either LQTS or HCM, recent SCD of a relative) entered at step 1 of the model accounted for 10% of the variance in general anxiety, with gender (p < 0.01) and recent SCD (p = 0.04) as significant predictors. After entry of the symptoms of heart-focused anxiety (avoidance, attention, and fear) at step 2 the total variance explained by the model was 33%, F (8, 114) = 7.06, p < 0.01. The symptoms of heart-focused anxiety uniquely explained 23% of the variance in general anxiety, R squared change = 0.23, F change (3,114) = 13.3, p < 0.01. In the final model, gender, recent SCD in the family, avoidance, and fear were statistical significant, with the fear scale reporting the highest beta value (beta = 0.32, p < 0.01).

**Table 4:** Hierarchical regression analyses assessing cardio-protective avoidance (avoidance), heart-focused attention (attention) and fear about heart sensations (fear) (CAQ)(scores 0-4) as independent determinants of general anxiety, depression (HADS) (scores 0-21), and physical health (PCS) (scores 0-100).

	Variables	R^2 change^	B	SE B	β	p-value
Step 1	**HADS Anxiety**	0.10				
	Male gender		-2.07	0.73	-0.26	**<0.01**
	Age		-0.002	0.03	-0.01	0.94
	Children		0.29	1.14	0.03	0.80
	Diagnosis		0.90	0.84	0.10	0.29
	Recent SCD in the family		1.93	0.92	0.19	**0.04**
Step 2						
	Male gender	0.23	-1.57	0.64	-0.20	**0.02**
	Recent SCD in the family		1.79	0.80	0.18	**0.03**
	Avoidance		1.09	0.45	0.22	**0.02**
	Attention		0.40	0.62	0.07	0.52
	Fear		1.65	0.54	0.32	**<0.01**
						
Step 1	**HADS Depression**	0.13				
	Gender		-0.70	0.66	-0.09	0.29
	Age		0.05	0.03	0.20	0.09
	Children		0.83	1.04	0.09	0.43
	Diagnosis		0.71	0.77	0.08	0.36
	Recent SCD in the family		1.83	0.84	0.20	**0.03**
Step 2		0.13				
	Recent SCD in the family		1.72	0.79	0.18	**0.03**
	Avoidance		0.93	0.45	0.21	**0.04**
	Attention		-0.45	0.61	-0.08	0.46
	Fear		1.42	0.53	0.30	**<0.01**
						
Step 1	**PCS Physical health**	0.24				
	Male gender		2.23	1.46	0.13	0.13
	Age		-0.22	0.06	-0.41	**<0.01**
	Children		2.19	2.30	0.10	0.34
	Diagnosis		-6.47	1.70	-0.33	**<0.01**
	Recent SCD in the family		0.69	1.84	0.03	0.71
Step 2		19.4				
	Age		-0.15	0.05	-0.28	**<0.01**
	Diagnosis		-4.29	1.57	-0.22	**<0.01**
	Avoidance		-4.00	0.92	-0.38	**<0.01**
	Attention		2.15	1.25	0.17	0.09
	Fear		-2.89	1.09	-0.26	**<0.01**

*Abbrev: *B: unstandardized coefficients; SE: Standard error; β: standardized regression coefficients; R^2 change^: determination coefficient change

HADS: Hospital Anxiety and depression scale; PCS: SF-36 Physical Component Summary; Diagnosis: Clinical diagnosis of Long QT syndrome or Hypertrophic cardiomyopathy; SCD: Sudden Cardiac Death

For the depression scale, the control variables in step 1 accounted for 13% of the variance in depression. Recent SCD in the family was the only variable significant (p = 0.03). After entry of the symptoms of heart-focused anxiety (avoidance, attention, and fear) at step 2, the model as a whole explained 25.8%, F (8, 114) = 5.0, p < 0.01. Apart from the variables controlled for, the symptoms of heart-focused anxiety explained an additional 13% in depression, R squared change = 0.13, F change (3, 114) = 6.7, p < 0.01. In the final model the following variables were significant; recent SCD in the family, avoidance, and fear, with the fear scale reporting a higher value (beta = 0.30, p < 0.01).

With regard to physical health, the variables (gender, age, having children, clinical diagnosis of either LQTS or HCM, recent SCD in the family) entered at step 1 of the model accounted for 24% of the variance in physical health. Increasing age (p < 0.01) and clinical diagnosis of either LQTS or HCM (p < 0.01) were significant predictors. After entry of the symptoms of heart-focused anxiety (avoidance, attention, and fear) at step 2 the total variance explained by the model was 44%, F (8, 109) = 10.6, p < 0.01. The symptoms of heart-focused anxiety explained an additional 19% in physical health after controlling for step 1 variables, R squared change = 0.19, F change (3,109) = 12.5, p < 0.01. In the final model age, clinical diagnosis, avoidance, and fear were statistical significant, with the avoidance scale reporting the highest beta value (-0.38, p < 0.01).

## Discussion

The present study aimed to investigate the role of three distinct symptoms of heart-focused anxiety (avoidance, attention, and fear), in relation to general anxiety, depression and physical health in patients referred to cardio-genetic counseling and at higher risk than the average person for serious arrhythmias and SCD because of a personal or a family history of an inherited cardiac disorder (familial LQTS or familial HCM). First, the levels of general anxiety, depression, and physical health in the patients were investigated in comparison to expected scores of the general population, further, the scores of general anxiety, depression, physical health, and heart-focused anxiety of patients with familial LQTS were compared to the scores of patients with familial HCM, and finally, the independent influence of avoidance, attention, and fear were examined in relation to general anxiety, depression, and physical health.

### General anxiety, depression, physical health, and heart-focused anxiety

High levels of general anxiety may be one of the major psychological problems among patients referred to genetic counseling for inherited cardiac disorders. In this study, approximately one quarter of the sample had clinical anxiety symptoms, whereas 13.5% scored above cut-off for depression. Further, the mean general anxiety scores were found to be significantly higher in the patients as compared to expected scores of the general population, whereas there were no significant differences in depression scores. The same pattern was found when analyzing patients at genetic risk in comparison to expected scores, this in contrast to previous findings among HCM mutation carriers without manifest disease [11], and in LQTS carriers without abnormal ECG [12]. In the previous reports, baseline data (i.e. before the patients had attended genetic counseling) were not analyzed, as in the present study, which may have biased their findings [11,12]. In addition, when comparing general anxiety and depression scores in patients with familial LQTS to familial HCM no significant differences were found, which was somewhat unexpected since previous research on patients with familial HCM especially has identified poor health-related quality of life in both mental an physical domains [16]. This may suggest that the overall level of general anxiety before receiving genetic counseling is determined by other factors than disease status or the inherited cardiac disorder in question in the present sample. Heart-focused anxiety may be one of the important reasons for elevated general anxiety in patients referred to genetic investigation and counseling for familial LQTS or familial HCM. Common for the patients with familial LQTS and familial HCM receiving genetic investigation and counseling, is a disease threat that runs in the family. The patients may have experienced other family members' disease or have a family history of SCD, factors that are known to cause high levels of heart-focused anxiety [31]. For example, in the present sample 28% of the patients had experienced a sudden cardiac death in a first or second degree relative. Patients with this experience and patients uncertain whether other relatives had undergone genetic testing had higher levels of heart-focused anxiety up to one year after genetic counseling, whereas satisfaction with the procedural parts of genetic counseling predicted decreased levels of heart-focused anxiety over time [23]. Therefore, it is possible that satisfaction with genetic counseling also will lead to decreased levels of general anxiety, however the different subgroups may show different patterns with that, as previous research has indicated[11,12].

In the present study, patient-reported physical health was overall as expected in a general population. However, physical health differed according to disease status. As expected, patients with a clinical diagnosis of either LQTS or HCM reported poorer physical health compared to expected scores of the general population, whereas patients at genetic risk reported somewhat better physical health. The manifestation of cardiac symptoms may be more likely in the group that already have been diagnosed, especially among the patients with HCM, who in addition to the risk of arrhythmias, can experience quite debilitating cardiac symptoms, which is the most likely explanation of the poorer physical health reported. This was further supported in the subgroup analyses. However, patients at genetic risk also have a substantial risk of having inherited the condition (50% for first-degree relatives), and thus a significant risk that sensations and stimuli from the heart can be potentially life threatening, which is a possible explanation why they presented with similar elevated general anxiety levels as the patients with a clinical diagnosis. However, we did not expect that they would report better physical health than the general population, but this phenomenon has been previously observed in similar populations [11], and may be caused by confounding factors such as younger age in the sample as compared to that of the norm population, or again, the experience of family members' illness, or SCD may cause individuals to value their own health more.

### Symptoms of heart-focused anxiety independently related to general anxiety, depression, and physical health

To address the overall aim of the study, the question posed is to what extent levels of general anxiety, depression, and physical health could have been independently influenced by the three distinct symptoms of heart-focused anxiety (avoidance, attention, and fear).

Partially consistent with the hypothesis, the result showed that avoidance and fear were symptoms of heart-focused anxiety that were significantly related to general anxiety, depression, and physical health, but attention was not. Specifically, patients who had higher levels of avoidance and fear were more likely to report higher levels of general anxiety, depression, and poorer physical health. A somewhat larger effect was observed for fear compared to avoidance in predicting general anxiety and depression, whereas avoidance had a stronger association to physical health.

The interpretation of the role of avoidance is not straight forward. Cardio-protective avoidance has been described as one of the cardinal symptoms of heart-focused anxiety [17,32,33]. Besides the fact that our findings showed that avoidance was uniquely related to general anxiety and depression, it is in fact one of the recommendations to this patient group [8]. "Cardio-protective avoidance" may equate to good patient adherence to appropriate medical recommendations. That is, a patient who has been given a diagnosis or are at genetic risk of either LQTS or HCM will often be coached to avoiding competitive athletic activity to prevent arrhythmia or sudden cardiac death, as cardio- protective avoidance. Avoidance of such activities does in that case not signify fearful symptoms, rather, appropriate adjustments to the limitations imposed by the disease, in line with our finding that higher avoidance scores is strongly related to poorer physical health, beyond the effects of gender, age, having children, clinical diagnosis and a recent SCD of a relative, as well as fear and attention. Interestingly, even if avoidance may be perceived as a an adaptive coping response or as a preventive measure, avoidance is also strongly related to higher levels of general anxiety and depression, which indicates that avoidance includes more than being an adaptive coping response. Thus, the current findings suggest that avoidance may be part of a psychological process highly influential in the production of general anxiety and depression, in addition to its relation to poorer patient-reported physical health.

This has implications for the genetic counseling of these patients. By addressing avoidance in the patient, the counselor will have access to important information to target intervention, and can provide information about that normal activities are not harmful. This may be important to prevent a vicious circle for the patients since avoidance may disrupt not only physical activity but also social life and occupational life functioning if such avoidance escalates [33]. In predicting avoidance, a mutation negative result was related to decreased avoidance [23]. Information about consequences of genetic testing may therefore be of influence, since a mutation positive test result will emphasize certain activity restrictions, while a negative more or less can rule out the recommendations.

Addressing fear of SCD is according to genetic counseling literature the main concern of psychological counseling in LQTS and HCM patients [13]. Results from the hierarchical regression analyses show that besides from significant effects of recent SCD in the family and cardio-protective avoidance, fear about heart sensations is the symptom that is strongest associated to general anxiety and depression, giving support to that this is a concern also to be reckoned with in genetic counseling of these patients. This tally also with previous research which found that perceived risk of SCD were associated with higher levels of general anxiety, depression and poorer physical health and that perceived risk of symptoms were associated with impaired mental health [11].

In contrast to the two other symptoms, heart- focused attention did not make a unique contribution in explaining the health outcomes even if attention was strongly correlated to general anxiety in particular. This may be due to the high intercorrelation with fear. Finally, gender, age, presence of a diagnosis of LQTS or HCM, and a relative's recent SCD, made significant contributions to the final models. In line with research of anxiety and gender, male gender was associated to less anxiety [34]. Not surprisingly, increasing age and clinical diagnosis of either LQTS or HCM was related to poorer physical health, and finally, a recent SCD in the family was related to higher levels of general anxiety and depression.

### Study limitations and strenghts

The design of this study shares the limitations that all cross-sectional designs have regarding control, causality and generalizability. Our sample size was relatively small, but the data was, however, collected at three different hospitals in three different health regions of Norway to reduce possible influence of community characteristics. The two patient groups (i.e. patients with familial LQTS and patients with familial HCM) differ from each other in some characteristics; however in the genetic counseling setting it is interesting to analyze them together since they are very similar with regard to the risk they are living with, they share some common disease manifestations, and LQTS and HCM are both autosomal dominant disorders with variable penetrance and disease expression. An important issue in discussing the findings in the present study is whether the research sample is representative of a greater population and what kind of biases might influence the results. Ideally we would like to generalize the findings in our study to all subjects undergoing genetic counseling for LQTS and HCM. The proportion of decliners in the study was 26.6%. The Regional Committee for Medical and Health Research Ethics did not allow collecting information for individuals who did not consent to research. Therefore, it was not possible to compare respondents from non-respondents. The comparison of general anxiety and depression scores with expected scores of the general population was a clear strength of study, since assessing symptoms based only on cut-off points may be of little clinical significance.

Finally, the study group consisted of both patients with a clinical diagnosis and patients at genetic risk, which can be regarded as very different groups. However, controlling for this in the analyses showed that this was meaningful for the study's findings, as it was confirmed that being clinically affected only had a significant relationship to patient-reported physical health, whereas it did not relate to levels of general anxiety and depression.

## Conclusion

In summary, the present study demonstrated higher general anxiety levels among the patients compared to expected scores of the general population. One fourth of the patients were clinically anxious, and 28% of the patients had experience of SCD among first or second degree relatives, 20% of the patients as recent as in the last year. General anxiety and depression levels seemed to be unrelated to having a clinical diagnosis. A more likely reason for the raised general anxiety level may be that living with the genetic risk of a life-threatening disorder and the uncertainty regarding cardiac symptoms causes raised levels of general anxiety, especially in patients with higher levels of heart-focused anxiety. Supporting our hypothesis, it was found that cardio -protective avoidance and fear about heart sensations may be part of a psychological process that appear to raise levels of general anxiety, depression, in addition to that it is related to poorer patient-reported physical health.

The prediction of risk, information of treatment strategies and preventive measures is well established in the genetic counseling method. This finding might therefore be of particularly clinical interest since it might strengthen the message that the genetic counseling of inherited cardiac disorders should be optimized with respect to not only helping the patients reducing danger of heart-related events by identifying who is at risk, but balancing it with the motive of helping the patients to manage *avoidance *behavior to minimize cardiac symptoms or complications, increased levels of heart-focused *attention *and monitoring of cardiac related stimuli, and *fear *and worries about heart-sensations and functioning. The possibilities of genetic testing can give more certainty as to that their perception of health is accurate, and counseling can influence more adaptive coping responses and health outcome. Future research should explore further whether factors related to genetic investigation influences symptoms of heart-focused anxiety.

## Competing interests

The authors declare that they have no competing interests.

## Authors' contributions

AH has taken main responsibility for the study's data collection, analyses, interpretation of the results, and in writing the first draft. AH has been the corresponding author. NØ participated in the preparation and conduct of the study and the editing of the article. BR contributed to shaping of the article and the editing of the article. GEE contributed to the statistical analyses and the editing of the manuscripts. KN participated in preparation and the editing of the article. All authors have read and approved the final manuscript.
